# Verifying the accuracy of statistical significance testing in Campbell Collaboration systematic reviews through the use of the R package *statcheck*

**DOI:** 10.4073/csrm.2018.1

**Published:** 2018-12-14

**Authors:** Joshua R. Polanin, Michèle B. Nuijten

**Affiliations:** ^1^ American Institutes for Research USA; ^2^ Tilburg University The Netherlands

## Introduction

### 1.1 Background

The synthesis of effect sizes using meta‐analysis does not require the use of null hypothesis significance tests (NHST). A meta‐analyst may combine effect sizes from multiple primary studies using a model that calculates the weighted average of the treatment effect, report the statistical precision of that estimate via a confidence interval (CI), and estimate the heterogeneity of those effect sizes—all without the need to report a test statistic or *p*‐value.

Nevertheless, the use of statistical significance testing in meta‐analysis is high (Polanin, 2013). A recent review of meta‐analyses published in the social sciences (Polanin & Pigott, 2015) revealed that the average review involved nearly 60 NHSTs. Most statistical significance tests resulted from tests of heterogeneity, and many were from tests of the overall average effect.

Because these significance tests can lead to policy and practice decisions, their accuracy is paramount. A recent review of significance testing in primary studies found, however, that one in eight primary studies published in eight high‐profile psychology journals had “grossly inconsistent *p‐*values that may have affected the statistical conclusion” (Nuijten, Hartgerink, Van Assen, Epskamp, & Wicherts, 2016, p. 1205). The authors applied the phrase “grossly inconsistent” to represent the occurrence of a *p*‐value error where the conclusions of the significance test would change based on a recalculation of the *p*‐value (i.e. a study's author said a *p*‐value was < .05 but the test statistic and degrees of freedom indicated the *p*‐value was actually > .05 or vice versa). In other words, an alarmingly high number of impactful results of statistical significance tests were inconsistent and misleadingly inaccurate:the gross inconsistencies indicated a systematic bias toward reporting statistically significant results.

NHSTs in primary research and the use of the associated *p*‐value is increasingly controversial. The academic journal *Basic and Applied Social Psychology* banned the reporting of NHSTs (Trafimow & Marks, 2015). Bayesian statisticians recommend complete removal of NHST from scientific research (see e.g. Wagenmakers, 2007). And the statistical journalism Website *FiveThirtyEight*(www.fivethirtyeight.com) published several pieces on researchers and statisticians’ abuse and misunderstanding of the *p*‐value (Aschwanden, 2016). These discussions, along with many others, culminated in 72 prominent statisticians declaring that the threshold for statistically significant *p*‐values should be lowered to < .005 (Benjamin et al., 2018; Lakens et al., 2018).

Given the overlap of content areas across the social sciences, it is likely that Campbell systematic reviews and review authors rely similarly on tests of statistical significance. To maintain Campbell'slevel of reliability, transparency, and excellence, it is imperative that NHSTs are reported accurately and consistently.

### 1.2 Research statements

Despite increased interest in NHSTsamong the primary research community, discussion of statistical significance testing in meta‐analysis remains infrequent. It is also impractical to suggest the complete removal of NHST from meta‐analyses. Moreover, it is likely researchers’ use of NHSTs will continue.

Therefore, the purpose of this project was to further elucidate statistical significance testing in meta‐analysis. We conducted this project with five objectives in mind:

1. To examine how meta‐analytic statistical significance tests are reported and discussed in Campbell reviews.
2. To modify the R package *statcheck* (Epskamp & Nuijten, 2016) to automatically extract statistical significance tests reported in‐text (as opposed to tables; discussed below) of published studies by meta‐analysis authors.
3. To evaluate the accuracy of statistical significance tests reported in published Campbell reviews using the adapted *statcheck* package.
4. To compare (a) the frequency of statistical significance tests that adhere to the *Publication Manual of the American Psychological Association* (APA; 2010) reported in reviews conducted across multiple research fields and (b) the accuracy of these reported tests using the adapted *statcheck* package across multiple fields (i.e. Campbell reviews, education and psychology reviews, and interdisciplinary reviews about IQ and intelligence).
5. To create a Campbell editorial brief to guide review authors on how to report statistical significance tests in‐text and to provide a tutorial for using the Web‐based *statcheck* program (statcheck.io).

### 1.3 The R package *statcheck*

To conduct this study, we focused on the use of the R package *statcheck* (Epskamp& Nuijten, 2016),which is a free, open‐source program that scans articles to locate and extract NHSTs reported in‐text in APA Style (e.g. *t*(28) = 0.32, *p* = .751; APA, 2010). The program then recalculates the *p*‐value based on the reported test statistic and degrees of freedom to check if the result is internally consistent. We use the word consistent here to mean that the reported *p*‐value matches the *p*‐value recalculated using the reported test statistic and degrees of freedom. This information can then be summarized across a range of studies automatically (for details, see Nuijten et al., 2016). More package details and information on how to download it can be found on the CRAN package page (https://cran.r‐project.org/web/packages/statcheck/index.html).

The *statcheck*R package has shown a lot of potential so far. It is used in the peer‐review process of the journal *Psychological Science* and the *Journal of Experimental Social Psychology*. It has been downloaded over 8,400 times, and the Web applicationstatcheck.io has been visited over 36,400 times since its launch in September 2016. And, *statcheck*'s benefits have been discussed in *Nature* (Baker, 2015, 2016; Nature, 2016), *Science* (Singh Chawla, 2017), *The Guardian* (Buranyi, 2017), and other media outlets. Furthermore, an additional validity study confirmed statcheck's accuracy (Nuijten et al., 2017; preprint).

Authors can report the results of statistical significance testing in two ways:

1. In‐text, where the authors report the statistical significance test in sentence form.
2. Ina table, where the statistical significance test is reported in a table.

This report, and *statcheck* in general, focuses on the first type of reporting for two reasons:

1. Authors who report the results of statistical significance testing in‐text are, implicitly or explicitly, highlighting specific test results. Although Campbell reviews do not have page limits, it is reasonable to suspect that review authors have little incentive to discuss each and every statistical significance test they conducted. Therefore, it is reasonable to assume the statistical significance tests that they reported in‐text are important.
2. In a strictly practical sense, *statcheck* was devised and refined for the investigation of statistical significance tests in‐text. Adapting it to investigate tests within tables would have required significant reprogramming and may not even been possible. Taken together, for theoretical as well as practical reasons, we focused exclusively on statistical significance tests reported in‐text.

### 1.4 How statistical significance tests are reported in published Campbell reviews

Currently, Campbell review authors who wish to write and report on the statistical significance tests they conducted have limited editorial or methodological guidance from Campbell policy documents. The pertinent section in the *Campbell Collaboration Systematic Reviews: Policy and Guidelines* lacks direction on how to report tests of statistical significance: “The review should present point estimates for mean effect sizes with their corresponding CIs (and prediction intervals, if appropriate) for each outcome of interest, and include measures of variability and heterogeneity (τ2, I2, or Q)” (Steering Group of the Campbell Collaboration, 2014, p. 40). Thus, one of our primary goals of this project was to determine whether review authors reported the results of statistical significance tests in a constant manner and, if not, how review authors currently reported their results.

To understand how review authors historically represented their results, we reviewed and manually extracted (i.e. not through the use of *statcheck*) the in‐text statistical significance tests reported in Campbell reviews. From the library of 135 Campbell reviews publicly available during the week of 22 May 2017, we selected 23 for review. We first randomly selected four reports for review. Then each author reviewed 9 or 10 reports. We sorted the reports based on the first authors’ last name, then we worked from the top of the alphabet and the bottom of the alphabet simultaneously.^1^

Of the 23 reviews initially selected, 20 conducted a quantitative meta‐analysis, and of those, 17 reported the results of the meta‐analysis in‐text. From the 17 review articles, we manually extracted all statistical tests reported in‐text. We did not, however, extract references to parameter estimates, such as when an author reports the tau‐squared, or the weighted average only, without a 95% CI or *p*‐value. We then classified the tests into three categories:

1. Point estimate and CI only;
2. Confidence interval that includes the discussion of statistically significance or the reporting of a statistical significance test; or
3. Report of statistical significance test only.

Across the 17 Campbell reviews, review authors reported 492 in‐text statistical significance tests. The average review in our sample reported 28.91 statistical significance tests (standard deviation [SD] = 27.45, 95% CI15.89 to 41.99). However, 102 of those 492 (20.73%) extracted results reported on the point estimate and 95% CI and did not discuss statistical significance or report the results of a statistical significance test. For strict statistical significance test reporting, therefore, the average review conducted and reported 23.64 tests (SD = 24.66, 95% CI11.93 to 35.37). The average review reported 4.82 statistical significance tests with a test statistic or *p*‐value (SD = 6.78, 95% CI1.60 to 8.05). Whichever way we disaggregate the results, it is clear that review authors rely on and report the results of statistical significance tests.

Our primary concern, however, was not the frequency of in‐text statistical significance testing. As such, we next sought to understand how review authors reported the results of statistical significance tests and whether they reported them with any consistency. To do so, we categorized the reported statistical significance tests by the type of test conducted and how the results were presented (i.e. CI only, CI with discussion of significance, or significance test only). We categorized the type of test into seven groups:

1. Tests of the average effect size standardized mean‐difference, either *d*, *g*, or undescribed (*n* = 235 [47.7%]);
2. Odds ratio (*n* = 115 [23.4%]);
3. Rate ratio (*n* = 49 [10.0%]);
4. Tests of the heterogeneity of effect sizes chi‐square tests (*n* = 57 [11.6%]);
5. Omnibus Q tests (*n* = 17 [3.5%]);
6. Explanations of heterogeneity Q‐between (*n* = 3 [0.006%]); and
7. Meta‐regression coefficients (*n* = 6 [0.01%]).

To demonstrate the myriad ways review authors present the results of statistical significance tests, we provide a sampling of their reporting below. We focused on the two presentations of results that included a discussion of statistical significance, because in‐text references to the 95% confidence, without discussing the “significance” of the effect or its overlap, does not technically qualify as a statistical significance test.

#### 1.4.1 Tests of the average effect size

Campbell review authors do not report the results of NHST consistently across reviews. Some discuss the point estimate in‐text and then list the 95% CI parenthetically. Others write out the words “confidence interval” in the parenthetical statement. Still others report the point estimate and 95% CI in the parenthetical statement but do not follow APA Style guidelines. Some examples of the differences in reporting style are given in the following section.

*1.4.1a Confidence interval with a discussion of significance or inclusion of statistical test*

“The overall effect size mean is 0.48 with a 95 percent confidence interval that covers zero

(‐0.36, 1.31)” (Spier et al., 2016, p. 40).

“Both studies found a small increase in crime with an overall mean percent change of 9.5% (95% confidence interval of ‐9.1% to 24.9%, p = 0.292)” (Wilson, Gill, Oladhere, & McClure, 2016, p. 29).

*1.4.1b Statistical significance test results only (no confidence interval reported)*

“The pooled results indicate an overall moderate, positive effect (d = .503, p = .03; Tables 8 and 9)” (Zief, Lauver, & Maynard, 2006, p. 14).

“For the 4 night only studies, the RES was 1.01 (n.s.), indicating no effect on crime” (Welsh & Farrington, 2008, p. 18).

#### 1.4.2 Tests of the heterogeneity of effect sizes

The tests of heterogeneity of effect sizes and the accompanying explanations of heterogeneity are described below. Relative to the NHST of tests of average effects, review authors’ reported tests of heterogeneity follow a more consistent framework, whereby the review authors generally report the test statistic, degrees of freedom, and *p*‐value. Similar to the tests of average effects, however, review authors do not follow APA Style guidelines, and, moreover, across Campbell reviews the NHST results reported in a variety of ways.

*1.4.1a Confidence interval with a discussion of significance or inclusion of statistical test*

No tests of heterogeneity appeared with a CI and discussion of a statistical test.

*1.4.2b Statistical significance test results only (no confidence interval reported)*

“The 18 studies were significantly heterogeneous according to the Q statistic (Q=35.72, 17 d.f., p<.0001)” (Bennett, Holloway & Farrington, 2008, p. 28).

“The test of heterogeneity was not significant for this outcome (P = 0.08; I² = 56%; Tau² = 0.33; Chi²= 6.80)” (Winokur, Holtan, & Batchelder, 2014, p. 33).

#### 1.4.3 Explanations of heterogeneity

Across our sampling of Campbell reviews, review authors did not report many NHST results from these tests. We expect that this trend will discontinue as Campbell review authors become more sophisticated in their analyses and as reviews continue to grow in primary study sample size. Moreover, from our limited sampling of statements, it is clear that review authors report the NHST results in a variety of ways.

*1.4.3a Confidence interval with a discussion of significance or inclusion of statistical test*

“Not surprisingly, the rape myths acceptance outcome had significantly larger effects (β = ‐.44, 95% C.I. = ‐.84, ‐.05)” (De La Rue, Polanin, Espelage, & Pigott, 2014, p. 93).

“Meta‐analysis of data from seven studies revealed a statistically significant difference between intervention and control conditions favouring the intervention (SMD ‐0.32, 95% CI ‐0.63 to ‐0.01, P = 0.04, n = 636, Analysis 1.7.2) but with evidence of significant heterogeneity (I2 = 68%; P = 0.005)” (Barlow, Smailagic, Huband, Roloff, & Bennett, 2012, p. 36).

*1.4.3b Statistical significance test results only (no confidence interval reported)*

“Furthermore, the addition of these moderators does little to change the moderating effect of the ‘intensity of conditionalities:’ the coefficient estimate for that variable is 1.08 (p‐value=0.005)” (Baird, Ferreira, Özler, & Woolcock, 2013, p. 39).

“For the treatment and catchments areas, the respective values of 34.8 (df=15) and 37.9 (df=15) were statistically significant at the p<0.05 level” (Bowers, Johnson, Guerette, Summers, & Poynton, 2011, p. 29).

#### 1.4.4 Conclusions drawn from sampling of statements

What is clear from this sample of statistical statements is that Campbell review authors vary in how they report the results of meta‐analytic NHST. Some present the CI within the sentence and then present additional information about the test statistic inside a parenthetical statement. Others discuss statistical significance without providing a test statistic; instead, they report the point estimate and CI. Still other authors provide all relevant statistical significance information within a parenthetical statement. And, a small minority of authors does not present any quantitative estimate of statistical significance and instead simply report that a finding is not statistically significant (i.e. “n.s.”).

### 1.5 Modifying the *statcheck* R package

What is also clear from the sample of statistical statements is that the current version of *statcheck* does not sufficiently account for meta‐analytic NHST. The current version will extract and analyze several types of statistical significance tests. These include tests of between‐group differences (*t*‐tests and *z*‐tests), goodness‐of‐fit tests (χ^2^), ANOVA‐based tests (*F*‐statistics), and tests of correlations (*r*). This means that some of the meta‐analytic NHST, such as the tests and explanations of heterogeneity, will not be extracted and analyzed. As a result, *statcheck* must be modified to reflect these findings.

To allow *statcheck* to account for tests that are more commonly used in meta‐analyses, we modified the current program to include *Q*‐based tests of heterogeneity. These include the omnibus *Q*‐test for heterogeneity of effects, the *Q*‐between test of between‐subgroup variance, and the *Q*‐within test for within‐subgroup variance. For example, a review author using an ANOVA‐based NHST of the difference between three subgroups should report the results as follows:

The results of the one‐way ANOVA‐like moderator test revealed that the age of the sample, either elementary, middle, or high school aged, statistically significantly moderated the average effect size (*Q_B_* (2) = 6.71, *p* = .03).

Appendix A illustrates this example as well as most of meta‐analytic NHST examples.

Finally, although *statcheck* has been updated to include these various tests, it will continue to function if and only if review authors report in‐text NHST results using APA Style reporting guidelines. Our recommended guidelines in Appendix A illustrate the use of the APA Style guidelines for reporting NHST. Following these guidelines will ensure consistent reporting across Campbell reviews.

### 1.6 Conclusions drawn from the modification of *statcheck*

One of the goals of this project was to investigate how Campbell review authors reported NHST results and to adapt the R package *statcheck* to account for this reporting. We found that Campbell review authors fail to report NHST using APA Style. Moreover, NHST results are reported in a variety of ways. We believe that providing the recommendations enumerated in Appendix A will help to correct this issue. Nevertheless, the results of our initial investigation also revealed that modifying *statcheck* to analyze the tests of heterogeneity would allow *statcheck* to be used by Campbell review authors, editors, and peer reviewers so long as APA Style guidelines are used.

What follows, then, is a larger investigation into the use and accuracy of meta‐analytic NHST within Campbell reviews as well as several other research fields. The modification of *statcheck* allowed us to complete these tasks.

## Methods

To answer research statements 3 and 4,^2^ we used the updated *statcheck* package to extract NHSTs from published meta‐analyses. The following describes our methodology.

### 2.1 Sample

Our primary sample included Campbell reviews published on or before 30 May 2017. As of that date, the Campbell review library included 135 publications. All publications were downloaded and included in the *statcheck* analysis.

We also included two additional samples in the analysis. The first derived from a review of social science meta‐analyses published in the *Review of Educational Research* and *Psychological Bulletin*. Polanin and Pigott (2015) used this sample of studies to investigate the prevalence of statistical significance tests conducted in meta‐analyses. To collect the review articles, we screened all journal citations for the presence of a quantitative synthesis of effect sizes, randomly samplinga total of 137 meta‐analytic articles.

The second sample derived from a review of the literature about intelligence and IQ (Nuijten, Van Assen, Augusteijn, Crompvoets, & Wicherts, 2018),in which the authors analyzed meta‐analyses found in ISI Web of Science as of August 2014 to estimate the typical effect size, the median power, and patterns of biases. They included quantitative meta‐analyses with complete data tables with sufficient information to calculate or retrieve primary study effect size and standard error. The final sample contained 130 articles.

### 2.2 Data collection

We extracted information about the number of statistical significance tests and their reported test statistics information with the tool *statcheck* (Epskamp & Nuijten, 2016),a free, open‐source R package that automatically extracts statistical results from papers and checks their internal consistency. One could compare it to a spellchecker for statistics (see Nuijten, 2018).

The *statcheck* tool looks for NHST results that are reported in‐text in APA Style. It recognizes *t*‐tests, *F*‐tests, *χ2*‐tests, *Z*‐tests, correlations and, from version 1.3.0 onward, it also recognizes tests of heterogeneity for meta‐analysis *Q*‐tests. These tests consist of three elements: a test statistic, degrees of freedom (except for Z‐tests), and a *p*‐value. These three elements are internally consistent: if you have two, you can calculate the third. The *statcheck* tool uses this characteristic to calculate whether an author‐reported *p*‐value matches the recalculated *p*‐value using the author‐reported test statistics.

The *statcheck* tool works as follows:

1. The program converts a PDF, HTML, or DOCX file to raw text. The program uses automated text search functions to search for APA Style–reported t‐tests, F‐tests, χ2‐tests, Z‐tests, correlations and Q‐tests. The program also extracts the symbol before the test statistics and *p*‐values because depending on what is reported, (=) or (< or >), the program modifies the statistical test. Different spacing has also been taken into account, and case is ignored.
2. The program then uses the reported test statistics and degrees of freedom to recalculate the *p*‐value.
3. Finally, the program compares the reported *p*‐value with the recalculated *p*‐value.
  a. Results flagged as “inconsistent” mean that the reported *p*‐value and the calculated *p*‐value do not exactly match.
  b. Results flagged as “grossly inconsistent” mean that that the reported *p*‐value and the calculated *p*‐value do not exactly match *and* the conclusions from the NHST would change.

Note that *statcheck* implicitly assumes that the *p*‐value is the inconsistent value, but it could just as well be the case that the test statistic or degrees of freedom contain a reporting error. The *statcheck* tool merely detects whether a set of numbers is consistent with each other.

In flagging results as consistent or not, correct rounding is taken into account. For instance, a reported *t* value of 2.35 could correspond to an actual value of 2.345 to 2.354, with a range of *p‐* values that can slightly deviate from the recomputed *p‐* value. The *statcheck* tool will not count cases like this as errors.

Furthermore, one‐sided testing is taken into account. If somewhere in the article the words “one‐tailed,” “one‐sided,” or “directional” are mentioned, and the result would have been correct if it was one‐sided, it is counted as a correctly reported one‐sided test.

The validity of *statcheck* was studied by comparing the results of *statcheck* with the results of a manual check of inconsistencies in a set of 49 primary studies in psychology. The *statcheck* tool detected about 60% of the NHST tests. Results that were not detected were usually reported in tables or not according to APA Style. In flagging inconsistencies and gross inconsistencies, the inter‐rater reliability between *statcheck* and the manual coding was .76 and .89, respectively (Appendix A in Nuijten et al., 2016). Furthermore, *statcheck*'ssensitivity (true positive rate) and specificity (true negative rate) were high: between 85.3% and 100%, and between 96.0% and 100%, respectively, depending on the assumptions and settings. The overall accuracy of *statcheck* ranged from 96.2% to 99.9% (Nuijten et al., 2017).

### 2.3 Analysis

Our analysis plan included a descriptive illustration using summary statistics. We did not plan to use or conduct inferential analyses. In addition to descriptive information, however, we also illustrated the proportion of reviews that included at least one APA Style–reported NHST in‐text over time. We graphed this information for easy visual inspection. We conducted all descriptive analyses using R and created the graphs using the R package *ggplot2* (Wickham & Francois, 2016).

## Results

### 3.1 Sample description

In total, we investigated 402 meta‐analyses. We downloaded and analyzed 135 Campbell reviews, and included 130 meta‐analyses^3^ about IQ and intelligence, and 137 meta‐analyses in the social sciences in general.

Approximately one in five (21.6%) meta‐analyses included at least one APA Style–reported in‐text NHST result (Table 1). This is a relatively low prevalence of NHST results, compared with the results of Nuijten et al. (2016) who found that about 60% of primary psychology articles contained APA Style–reported NHST results. Within each meta‐analysis, *statcheck* detected a median of 4 NHST results. Again, this is relatively low, compared to the approximately 10 results per primary psychology paper (Nuijten et al., 2016). See Table 1 for full details on the number of extracted APA Style–reported in‐text NHST results.

**Table 1:** General descriptive statistics of the number of meta‐analyses (MA) downloaded and APA style–reported in‐text NHST results they contained.

**Sample**	**# of MAs downloaded**	**# of MAs with APA Style–reported NHST results**	**% of MAs with APA Style–reported NHST results**	**# of APA Style–reported NHST results**	**Median # of APA Style–reported NHST results in‐text per MA**
**CC Reviews**	135	24	17.8	150	3.5
**IQ Reviews**	130	22	16.9	118	5.0
**Social Science**	137	41	29.9	268	4.0
**Total**	402	87	21.6	536	4.0

*Notes*: CC Reviews = Campbell reviews; IQ Reviews = a review of reviews in the general field of intelligence and IQ; Social Science Reviews = reviews from two leading social science review journals.

We also looked at the types of test most often reported. We found that the majority of the detected tests were Z‐tests (173), followed by F‐tests (97). Furthermore, we found 120 Q‐tests in total, of which there were 59 Q‐between tests and 4 Q‐within tests. For details, see Table 2.

**Table 2:** General descriptive statistics of the number of tests extracted from the full set of meta‐analyses.

**Type of statistical test**	**Number of results extracted**	**Proportion of Total**
*Z*	173	32.28
*t*	56	10.45
*F*	97	18.10
Chi‐square	76	14.18
*Q* (omnibus)	57	10.63
*Q‐*between	59	11.01
*Q‐*within	4	0.75
*r* (correlation)	14	2.61
**Total**	**543**	**100.00**

The proportion of NHST used by the review authors map onto those proportions reported in Polanin and Pigott (2015). In that study, the authors found that most of the NHST that authors reported were from tests of or explanations of heterogeneity.

### 3.2 Analysis of Campbell reviews

#### 3.2.1 General prevalence of APA Style–reportednull hypothesis significance test results

Twenty‐four of the 135 Campbell reviews (17.6%) we analyzed contained APA Style–reported NHST results in‐text. This is much lower than the general prevalence of APA Style–reported NHST results in primary studies (Nuijten et al., 2016). We found a median of 3.5 NHST results per meta‐analysis, which is also much lower than the approximately 10 results per primary study we found in previous research (Nuijten et al., 2016). See Table 1 for details.

**Figure 1:**
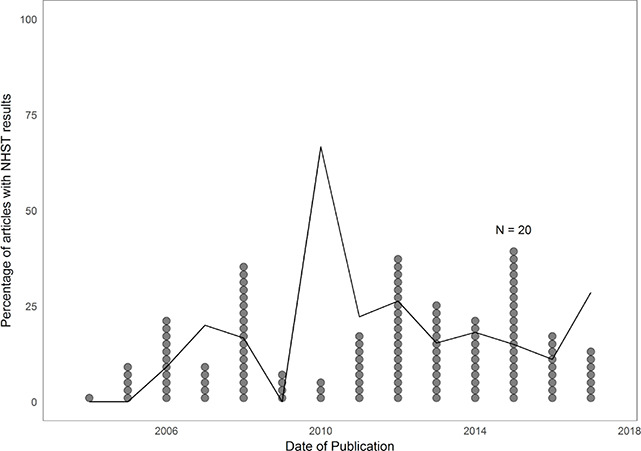
Overall prevalence of APA Style–reported NHST results in‐text in Campbell reports over time. The line indicates the proportion of articles with NHST results per year, and the number of dots per year indicate the total number of Campbell reports.

We looked at the prevalence of APA Style–reported NHST results in‐text in Campbell reports over time but did not find a clear trend (see Figure 1). In Figure 1, the line indicates the proportion of articles with NHST results per year, and the number of dots per year indicate the total number of downloaded Campbell reports. The more dots, the more Campbell reports and more reliable the percentage. For instance, in 2010 there seemed to be a spike in the prevalence of reports with NHST results, but this proportion is calculated based on only three reports. Taking the number of Campbell reports per year into account, the graph shows that the prevalence of APA Style–reported NHST results in‐text has been relatively stable over the years.

#### 3.2.2 General prevalence of null hypothesis significance test inconsistencies

We found that 10 of the 24 Campbell reports with APA Style–reported NHST results (41.7%) contained at least one inconsistent result. Two of the 24 reports (8.3%) contained at least one gross inconsistency, in which the recalculated *p*‐value showed a different statistical conclusion than the reported *p*‐value. The prevalence of these articles with at least one inconsistency or gross inconsistency is lower than those in primary studies (49.6% and 12.9%, respectively; Nuijten et al., 2016).

If we look at the prevalence of inconsistencies at the level of individual results, we find that of the 150 NHST results we extracted, 27 were inconsistent (18.0%) and 2 were grossly inconsistent (1.3%). This way of calculating prevalence of inconsistency does not take into account that results within a single article might be statistically dependent. Another way to estimate the inconsistency prevalence at the level of the individual resultsis to calculate the average percentage of inconsistencies within each article, and average these numbers across articles. If we do that, we find that on average 14.1% (SD = 24.2) of the results within a Campbell report are inconsistent and 4.8% (SD = 20.5) are grossly inconsistent. This prevalence is higher than in the individual primary studies (10.6%, and 1.6%, respectively; Nuijten et al., 2016). We further examine gross inconsistencies in the Discussion section below.

#### 3.2.3 Prevalence of inconsistencies per type of test

In our analyses, we also looked at the prevalence of statistical reporting inconsistencies per type of test. It is imaginable that some statistical tests are more difficult to report than others and, hence, more errorprone.

Table 3 shows the prevalence of inconsistencies in Campbell reviews per type of test. From these results it seems that chi‐square tests are most often reported inconsistently and grossly inconsistently (50% and 50%, respectively). These proportions are unreliable, however, because only 2 chi‐square tests were extracted. It seems that the general Q‐tests are particularly errorprone: 33.3% were inconsistently reported (and 16.7% of the Q‐between tests were inconsistently reported). Most of the flagged inconsistencies in the *Q*‐tests seemed to have been caused by incorrect rounding (e.g. reporting *p* = .411 instead of *p* = .410), possible typos (e.g. reporting *p* = .37 instead of *p* = .47), and not following APA Style reporting conventions (e.g. reporting *p* = .000 instead of *p*< .001).

**Table 3:** Prevalence of APA style–reported NHST results in‐text in Campbell reviews and their internal consistency per type of test.

**Type of statistical test**	**# of results extracted**	**# of inconsistencies**	**% of results inconsistent**	**# gross inconsistencies**	**% of results grossly inconsistent**
*Z*	60	9	15.0	1	1.7
*Q*	36	12	33.3	0	0.0
*F*	26	4	15.4	0	0.0
*t*	19	0	0.0	0	0.0
*Q‐*between	6	1	16.7	0	0.0
Chi‐square	2	1	50.0	1	50.0
*r*	1	0	0.0	0	0.0
**Total**	150	27	18.0	2	1.3

#### 3.2.4 Investigating systematic bias

Previous research found that gross inconsistencies were more prevalent in results reported as significant than results reported as nonsignificant, indicating evidence for a systematic bias toward reporting statistically significant results (Nuijten et al., 2016). We also investigated whether such a systematic bias was present in Campbell meta‐analyses. We found that of all results reported as significant, 1.22% were inconsistent. In results reported as nonsignificant, we found that 1.59% of the results were inconsistent. This means that we did not find a systematic bias toward reporting significant results in Campbell meta‐analyses.

### 3.3 Analysis of all reviews

#### 3.3.1 General prevalence of APA Style–reportednull hypothesis significance test results

Of the 402 meta‐analyses we analyzed in total (including Campbell reviews), 87 meta‐analyses contained APA Style–reported NHST results in‐text (21.6%). In total, we extracted 536 NHST results, which resulted in a median of 4.0 NHST results per meta‐analysis (see Table 1). The prevalence of APA Style–reported results in this sample of meta‐analyses is considerably lower than in a sample of primary psychology articles (Nuijten et al., 2016).

**Figure 2:**
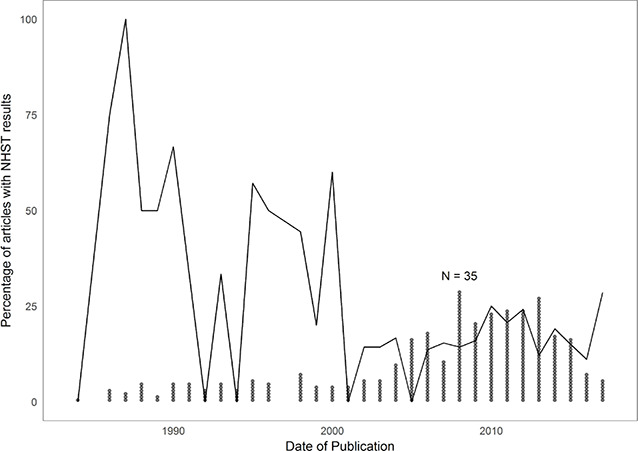
Overall prevalence of APA Style–reported NHST results in‐text over time. The line indicates the proportion of articles with NHST results per year, and the number of dots per year indicates the total number of downloaded meta‐analyses.

We inspected the trends over time in the prevalence of APA Style–reported NHST results in‐text, but the results indicated that the prevalence of APA Style–reported NHST in‐text remained relatively stable (see Figure 2). The line in Figure 2 indicates the proportion of articles with NHST results per year, and the number of dots per year indicates the total number of downloaded meta‐analyses. The lower the number of meta‐analyses in a year, the harder it is to interpret the proportion of meta‐analyses that contained NHST results. From 2000 onward, we had more meta‐analyses per year, and here we see a relatively stable trend in the proportion of meta‐analyses that contain APA Style–reported NHST results in‐text.

#### 3.3.2 General prevalence inconsistencies

We found that in total 34 of 87 (39.1%) meta‐analyses with at least one APA Style–reported NHST in‐text contained at least one inconsistently reported NHST result. Seven of the meta‐analyses contained a gross inconsistency (8.0%), in which the reported *p*‐value was significant and the recalculated *p*‐value was not, or vice versa. These numbers are lower than in primary research, where articles contained at least one inconsistency or gross inconsistency in 49.6% and 12.6% of the cases, respectively (Nuijten et al., 2016).

We also looked at the prevalence of reporting inconsistencies at the level of the individual results. We found that of 536 NHST results we extracted, 78 were inconsistent (14.6%) and 8 were grossly inconsistent (1.5%). These estimates do not take into account any statistical dependencies among NHST results within the same meta‐analysis, so we also calculated the percentage of inconsistent NHST results per meta‐analysis and averaged that over all meta‐analyses. We found that on average 14.8% (SD = 26.3) of the results in a meta‐analysis are inconsistently reported, and 2.1% (SD = 11.3) of the results in a meta‐analysis are grossly inconsistent (as compared with 10.6% and 1.6% in primary studies, respectively; Nuijten et al., 2016).

#### 3.3.3 Prevalence of inconsistencies per type of test

Looking at the full sample of meta‐analyses, we again see that the highest percentage of inconsistencies is detected in *Q*‐tests (28.1%). The *Q*‐between tests are also often inconsistently reported (16.9%). See Table 4 for details.

**Table 4:** Prevalence of APA style–reported NHST results in‐text in the full sample of meta‐analyses and their internal consistency per type of test.

**Type of statistical test**	**Number of results extracted**	**# inconsistencies**	**% of results inconsistent**	**# gross inconsistencies**	**% of results grossly inconsistent**
*Z*	173	20	11.6	3	1.7
*F*	97	14	14.4	0	0.0
Chi‐square	76	12	15.8	1	1.3
*Q‐*between	59	10	16.9	2	3.4
*Q* (omnibus)	57	16	28.1	0	0.0
*t*	56	4	7.1	2	3.6
*r*	14	2	14.3	0	0.0
*Q‐within*	4	0	0.0	0	0.0
***Total***	536	78	14.6	8	1.5

#### 3.3.4 Investigating systematic bias

In previous research it was found that gross inconsistencies were more prevalent in results reported as significant compared to results reported as nonsignificant, indicating evidence for a systematic bias toward reporting statistically significant results. We also investigated whether such a systematic bias was present in the meta‐analyses. We found that of all results reported as significant, 1.43% was inconsistent. In results reported as nonsignificant, we found that 1.83% of the results was inconsistent. This means that we did not find a systematic bias toward reporting significant results in this set of meta‐analyses.

## Discussion

The purpose of this study was to examine how NHSTs were reported and used by Campbell review authors, to modify *statcheck* so that it could be used by Campbell review authors as well as meta‐analysts in general, and then use the updated *statcheck* R package to investigate inconsistencies of in‐text meta‐analytic NHSTs.

Our analysis of Campbell reviews revealed that review authors rarely use APA Style reporting standards to report the results of meta‐analytic NHSTs. So we drafted an editorial brief (Appendix A) to guide Campbell review authors. After modifying the *statcheck* R package, the results of our analysis of meta‐analytic NHSTs indicated that review authors, both for Campbell and the social science field in general, reported a small but nontrivial percentage of inconsistencies, including gross inconsistencies. The results indicated that meta‐analysts, however, may report the results of NHSTs in a more accurate manner compared with previous work in primary research.

## 4.1 Interpretation of results

Our sample of studies included reviews published by Campbell, two high‐quality social science review journals, and an interdisciplinary set of reviews on IQ and intelligence from a variety of social science, medical, and other journals. The sample is far from random, given that the sampling frame is perhaps all social science reviews, and we did not select from that frame using any random procedure. Despite this, we feel confident that the results can be reasonably generalized to other reviews published in the social sciences.

Our interpretation of the field of reviews is that NHSTs are indeed used by review authors to report meta‐analytic results, only a small proportion report the results in‐text using APA Style reporting standards, and a nontrivial number of inconsistencies and gross inconsistencies persist.

Overall, we found a lower prevalence of meta‐analyses with at least one inconsistency or gross inconsistency than was found in primary research. However, at the level of individual NHST results, the prevalence of inconsistencies was higher in meta‐analyses than in primary studies. This discrepancy probably arises because meta‐analyses report fewer NHST results than primary studies.

Regardless of how we count the inconsistencies, it is important to comment on the eight total gross inconsistencies we found in seven independent meta‐analyses. We will not cite the specific articles from which the gross inconsistencies derived, because we feel that this would focus disproportionally on specific authors, whereas the problems we address are general. Table 5 lists all eight inconsistencies, the recalculated *p*‐values, and some brief comments on the findings.

**Table 5:** Gross inconsistencies reported by review authors.

Test	Reported Information	Re‐computed *p*‐value	Additional Information
1	*Q/Z* = 69.7, *p* = 0.404	.001	This was considered as a Z test, but it could be *Q* test.
2	2 (5, *n*=36) = 11.05, *p*< .05	.050	Reported incorrectly as less than .05.
3	*t*(24)= 1.77, *p*= .03	.089	Reported incorrectly as p = .03.
4	*Z* = 1.73, *p* = .04	.083	Possible one‐tail test, but no indication from authors.
5	*Q_b_* (1) = 3.78, *p*< .05	.051	Reported incorrectly as less than .05.
6	*t*(32) = 1.82, *p*< .05	.078	Possible one‐tail test, but no indication from authors.
7^*^	*Q_b_* (1) = 5.38, *ns*	.020	Reported an alpha of .01; correct interpretation.
8^*^	*Z* = 1.96, *ns*	.049	Reported an alpha of .01; correct interpretation.

* *Note:* *indicates test are from the same article; all reported information is taken directly from what the study authors reported in‐text.

The first gross inconsistencies represents improper APA Style reporting of the NHST. The test statistic and the *p*‐value did not align; here, the authors said clearly that the results were not statistically significant and yet, depending on the test, the results may have been statistically significant. Tests 4 and 6 could have possibly been conceived as one‐tail tests. Upon further examination of the reviews, however, the review authors did not say that any of the NHSTs was one‐tailed. It is likely that these three tests are either poor APA Style reporting or simply gross inconsistencies. Test 2 and 5 represent what we consider “classic” gross inconsistencies. The authors said that the *p*‐value was less than .05; yet upon recalculation, the *p*‐value was misrepresented.

The final two gross inconsistencies were flagged as inconsistencies but represent correctly reported NHSTs. By default, *statcheck* assumes an alpha of .05, which would mean the respective reported *p*‐values of .020 and .049 would actually be significant. However, the authors said their alpha level was 0.01 and the test statistics and their conclusions supported this representation. This example represents one of the problems with *statcheck*: taken over many studies, the system may improperly identify some NHSTs as gross inconsistencies. The opposite is likely true as well: some NHSTs reported as accurate may actually be inconsistent. We suggest that one should be careful when making conclusions about specific studies, unless the intended purpose is to examine a single study. In that case, the reader would do well to examine each and every gross inconsistency (or inconsistency, for that matter) to ensure that *statcheck* correctly identified any consistent or inconsistent results.

Finally, we did not find a systematic bias toward misreporting significant results. One theory to explain this has to do with the incentive structure assigned to meta‐analyses. Meta‐analysts are probably are not governed by the same incentives to which primary researchers adhere. Meta‐analysts’ future funding is not governed by the results of the statistical significance from a previous review. The editorial guidance given to peer reviewers or editors may not reflect the need to publish papers that illustrate significant findings. And, perhaps most importantly, meta‐analysts may not be driven to report statistically significant findings because their conclusions can, and should, be based on the magnitude of effects. The incentive structure to report statistically significant meta‐analytic results is simply not apparent. This hypothesis is potentially borne out in our results.

However: so long as meta‐analytic NHSTs are reported and used as the basis for conclusions, the field should ensure the accuracy of the tests. The modifications we made to *statcheck* ensure that an easy‐to‐use program is available.

## 4.2 Impact on Campbell reviews

The results of the review of in‐text statistical statements, collected both manually and by *statcheck*, indicated that Campbell review authors vary widely in the report of the results of NHSTs according to APA Style standards. In fact, although we did not investigate other reporting standards, we posit that Campbell review authors do not report the results of NHSTs using *any* reporting guidelines. Review authors may report NHST consistently within their reviews, but we found little consistency across reviews.

Lack of consistency in meta‐analytic NHST reporting on its own does not necessarily corrupt or otherwise diminish the important findings from these previous reviews. The lack of APA Style reporting does limit our ability to verify the accuracy of the reported NSHT statistics. Our manual findings, however, were confirmed by the data extracted from *statcheck*: Campbell review authors use meta‐analytic NHSTs and fail to report the results in an APA Style format.

As Campbell continues to grow and attract even greater visibility and reputability, we believe that it is imperative to ensure the accuracy of meta‐analytic NHST. Moreover, we believe that the best way to ensure the accuracy of NHST reporting is to use the modified *statcheck* Web application. And to do that, Campbell review authors must report the results of their meta‐analytic NHSTs using APA Style reporting standards.

The greatest impact of this study on Campbell reviews is the prescriptive reporting shift we suggest. Fortunately, our suggestion is easily implemented, given that APA Style is the standard for peer‐reviewed social science journals.

## 4.3 Impact on social science meta‐analyses

The impact of this study on social science meta‐analyses in general is much the same as on Campbell reviews. The extraction of NHSTs using *statcheck* revealed that, compared with previous work in primary research, meta‐analysts fail to report meta‐analytic NHSTs using APA Style reporting standards. For the same reasons that Campbell review authors should use APA Style guidelines, so should the field at large. The modified *statcheck* Web application and R package will work with any PDF, HTML, or DOCX file and should be used widely across the field.

Note that although our results did not reveal a large proportion of inconsistencies or gross inconsistencies, we did find inconsistencies nonetheless. Some baseline proportion of inconsistencies is perhaps unavoidable or understandable. Humans, not machines, transcribe the results of statistical analyses from the statistical applications to a word processor. Simple transcription errors are expected. The results may even indicate some level of comfort because meta‐analysts transcribe so few mistakes.

We believe, however, that our use of technology can dramatically decrease simple inaccuracies. The Web application *statcheck*, if used in the peer‐review process, should alert reviewers and authors to the prevalence of inconsistencies and the occasional gross inconsistency. Simply knowing that the to‐be‐submitted manuscript will be checked by *statcheck* may be enough to encourage meta‐analysts to double check their results and make any needed changes. We will communicate and encourage Campbell authors as well as social science review authors to use the *statcheck* Web application to ensure high‐quality NHST reporting.

## 4.4 Privacy concerns

The Web version of *statcheck* uses a browser‐based system in which the user uploads a PDF, HTML, or DOCX file to the program. After the program runs, the results appear on the webpage. Although the Web version of *statcheck* must inevitably interact with the article and its contents, the program does not store any of the extracted information on its server. As soon as the program has completed the analysis and the user closes the webpage, the information is removed from the server and is not stored. These concerns are also addressed on the FAQ page of the *statcheck* Web application (statcheckstatcheck.io/about.php). We take seriously researchers’ privacy concerns, especially regarding results from unpublished manuscripts.

## 4.5 Limitations

As we noted above, perhaps the biggest limitation of this study is the limited sampling frame. We did not have the capacity to estimate the sampling frame of all available published reviews and did not take a random sample from this sampling frame. Therefore, the results can be used to generalize to three specific populations:

1. Campbell reviews that report in‐text meta‐analytic NHST results using APA reporting style;
2. Social science reviews published in two high quality journals that report in‐text meta‐analytic NHST results using APA reporting style, or
3. Reviews that summarize IQ research and that report in‐text meta‐analytic NHST results using APA Style for reporting.

As we also argue above, we believe our sample represents the larger population of reviews. For all the reasons listed, however, we will refrain from placing too much emphasis on the field at large.

Also, this research is subject to unforeseen programming errors embedded within *statcheck* that prevent us from analyzing all in‐text APA Style–reported NHSTs. We spotchecked the extracted results, but we were not able to manually check all extracted results. However, two validity studies of the original version of *statcheck* extensively reviewed the outputs and found a relatively low number of false positives and false negatives in flagging inconsistencies and gross inconsistencies (see Appendix A in Nuijten et al., 2016; Nuijten et al., 2017). The *statcheck* tool's results will never be as accurate as a manual check, but when its limitations are considered, we can recommend *statcheck* for self‐checks, for use in peer review, and for research about the prevalence of inconsistencies in large samples of literature.

Finally, our results do not apply to the sometimes high number of NHSTs reported in manuscript tables. These results rarely, if ever, follow a particular pattern and are difficult to extract with any regularity. In the future, we may attempt to conform *statcheck* to accomplish this task. As it stands, we are limited to in‐text APA Style–reported results.

## 4.6 Conclusions

Our analysis of in‐text APA Style–reported meta‐analytic NHST results revealed that Campbell review authors, as well as social science review authors in general, use and report meta‐analytic NHSTs. Therefore, it is important to ensure that these results are accurate and reliably reported. The modification of *statcheck* to accommodate additional meta‐analytic NHST results will allow Campbell review authors to use *statcheck* and will help to ensure that results are reported accurately.

## Figures and Tables

**Figure A1:**
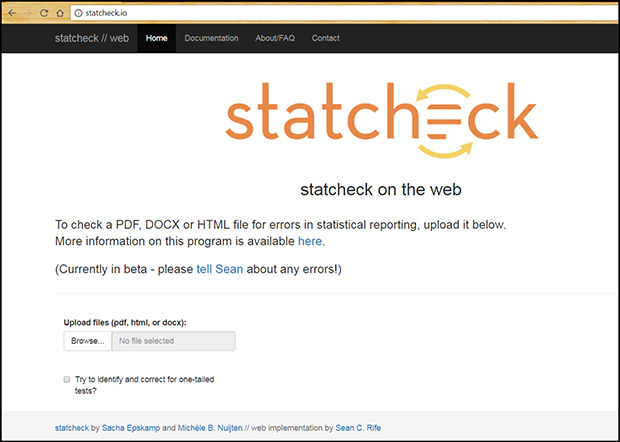
Screenshot of the Web application *statcheck*. To upload an article to scan, click on the “Browse…” button.

**Figure A2:**
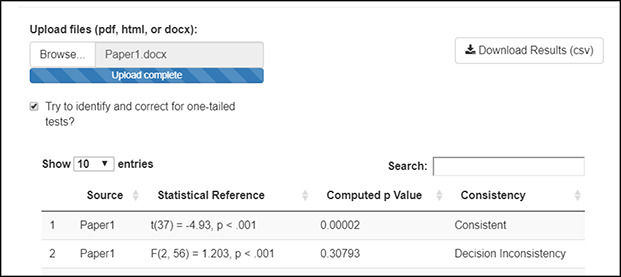
Screenshot of the *statcheck* results for a single uploaded article.

**Figure A3:**
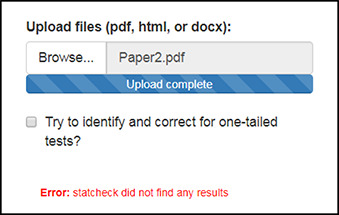
Screenshot of the output when *statcheck* did not detect APA Style–reported NHST results in the uploaded article.

**Figure A4:**
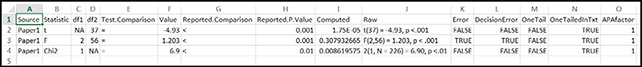
Screenshot of the downloaded results in CSV format.

**Figure A5:**
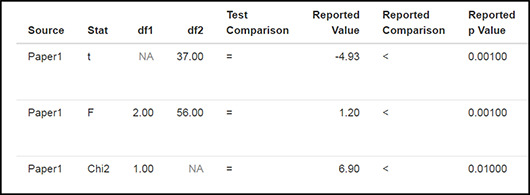
First part of the output that indicates information about which statistics are extracted.

**Figure A6:**
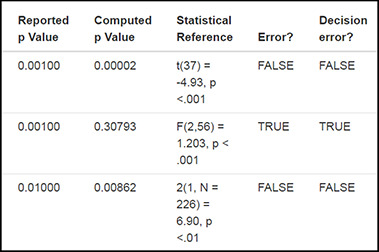
Second part of the output that indicates information about the internal consistency of the extracted result.

**Figure A7:**
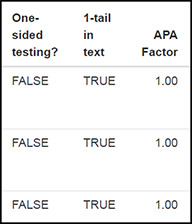
Third part of the output that indicates information about evidence that one‐sided tests were used and estimates what proportion of all results were reported in APA Style.

## Footnotes

1 Although this is not a strictly random set of studies, our goal was simply to review a set of studies to get a sense of how the results were reported. Our intention here was not to generalize to a wider population of studies.

2 To evaluate the accuracy of statistical significance tests reported in published Campbell reviews using the adapted statcheck package; and to compare a) the frequency of APA Style–reported statistical significance tests in reviews conducted across multiple research fields and b) the accuracy of these reported tests using the adapted statcheck package (Campbell reviews, education and psychology reviews, and interdisciplinary reviews about IQ and intelligence).

3 One article contained two independent meta‐analyses, but here we count it as a single article.

## Editorial brief

### Campbell editorial brief on reporting statistical significance tests in‐text

#### 1. Introduction

The *Campbell Collaboration Systematic Reviews: Policy and Guidelines* (The Steering Group of the Campbell Collaboration, 2014) document provides an excellent overview and explanation of the many standard practices Campbell review authors should use. What is lacking from that document, however, is an illustration of how to report the results of null hypothesis significance tests (NHST).
“The review should present point estimates for mean effect sizes with their corresponding confidence intervals (and prediction intervals, if appropriate) for each outcome of interest, and include measures of variability and heterogeneity (τ2, I2, or Q)” (p. 40).

Should review authors wish to conduct and report NHSTs, they do so without explicit guidance from Campbell'spolicy and guidelines documentation. Limited methodological policy or guidance can lead to underreporting, inaccuracy, or a lack of statistical transparency. As such, this editorial brief serves as a supplement to the *Campbell Collaboration Systematic Reviews: Policy and Guidelines* document and has two purposes:

1. To propose a set of editorial guidelines that can be used by review authors to create standardized, accurate, and transparent reporting of meta‐analytic statistical significance results within the written text using the *Publication Manual of the American Psychological Association* (APA; 2010) reporting standards.
2. To provide a brief guide to the *statcheck* Web application. The Web application automatically verifies the accuracy of statistical significance tests reported in‐text using APA Style reporting standards.

Notably, this brief and its authors do not advocate for (or against) meta‐analytic NHSTs. Empirical investigations into their use, however, has indicated that meta‐analysts use statistical significance testing at a high rate (Polanin & Pigott, 2015). Furthermore, as has been shown in primary research, statistical significance test results are prone to error and misrepresentation (Hoekstra, Finch, Kiers, & Johnson, 2006; Nuijten, Hartgerink, Van Assen, Epskamp, & Wicherts, 2016). If Campbell review authors are going to use such tests, they should know what to report and how to report them. This brief provides this critical knowledge.

#### 2. Standardized guidelines for reporting statistical significance tests in meta‐analysis

The following is an explanation of how Campbell review authors should report the results of meta‐analytic NHSTs. The guidance follows the logic set forth by APA (2010): “when reporting inferential statistics (e.g. t tests, F tests, χ^2^tests, and associated effect sizes and confidence intervals), include sufficient information to allow the reader to fully understand the analyses conducted” (p. 116). We have adapted these recommendations for NHSTs conducted by meta‐analysts.

##### 2.1. Overall main effect and subgroup analyses

Meta‐analytic average effect size statistical significance tests can be represented in two ways, depending on the model: *z* tests and *t* tests.

The *z* test is commonly reported and used in traditional meta‐analytic models. It may be reported with or without the point estimate and the 95% confidence interval (CI).
The results of the meta‐analytic model indicated a positive (d = .24, 95% CI .03 to .45) and statistically significant effect (z = 2.31, *p* = .02).

The *t* test is another commonly reported statistical significance test, especially given the rise in popularity of robust variance estimation. The regression model's results reported in *robumeta* (Fisher, Tipton, & Zhipeng, 2017) provides the *t* value as well as the associated degrees of freedom and exact *p*‐value.
The results of the meta‐analytic model indicated a positive (d = .24, 95% CI.03 to .45) and statistically significant effect (*t*(14) = 2.23, *p*= .04).

Regardless of the substantive nature of the average effect, either overall average or subgroup average, a discussion of the results of statistical significance testing should include appropriate test statistics, degrees of freedom, and exact p‐values.

#### 2.2. Identifying heterogeneity among effect sizes

A series of statistical significance testing follow standard practice meta‐analytic reporting. These statistical tests follow common practice among meta‐analysts to first test for heterogeneity among the effect sizes and then explain the heterogeneity.
Although the overall average effect size was not statistically significant, the results of the test for effect size heterogeneity revealed statistically significant heterogeneity (*Q*(14) = 24.64, *p* = .04).

#### 2.3. Explaining heterogeneity among effect sizes

The *Q* ‐ between test can be reported with a *Q_B_* or a *Q*‐between to represent the test statistic.
The results of the one‐way ANOVA‐like moderator test revealed that studies that the age of the sample, either elementary, middle, or high school aged, statistically significantly moderated the average effect size (*Q_B_* (2)= 6.71, *p* = .03). The results of the one‐way ANOVA‐like moderator test revealed that studies that the age of the sample, either elementary, middle, or high school aged, statistically significantly moderated the average effect size (Q‐between(2)= 6.71, *p* = .03).

Likewise, the *Q* ‐ within test can be reported with a *Q_W_* or a *Q* ‐ within.
Although age significantly moderated the average effect sizes, statistically significant variation within each level remained for two of the three levels: elementary (*Q_W_* (4) = 9.71, *p* = .05), middle (*Q_W_* (3) = 8.53, *p* = .04), and high school (*Q_W_* (8) = 10.12, *p* = .26). Although age significantly moderated the average effect sizes, statistically significant variation within each level remained for two of the three levels: elementary (Q‐within(4) = 9.71, *p* = .05), middle (Q‐within(3) = 8.53, *p* = .04), and high school (Q‐within (8) = 10.12, *p* = .26).

Given a significant *Q* ‐ between test, which signals significant between‐group variation, meta‐analysts may choose to test for differences between subgroups or moderator levels. This is akin to “post‐hoc” testing in primary research. Rarely, if ever, meta‐analysts adjust for multiple comparisons (Polanin, 2013). Regardless, the meta‐analyst should report the test statistic, degrees of freedom, and *p*‐value.
Studies that used a student‐informed measurement differed statistically significant from studies that used a teacher‐informed measurement (*t*(15) = 3.36, *p* = .004).

Continuous moderators or the desire to test multiple moderators simultaneously require the use of a meta‐regression model. The results of a meta‐regression model follow a *t* ‐ distribution (Tipton, 2015). To evaluate the test statistic and the associated statistical test, meta‐analysts should report the regression coefficient, its standard error, the test statistic, degrees of freedom, and exact *p*‐value.
The results of the meta‐regression model indicated that, controlling for five other variables in the model, the length of treatment in weeks was statistically significantly related to the size of the effect (*b* = .40, *SE* = .19, *t*(65) = 2.10, *p* = .04). Studies also explained a significant proportion of variance in effect size (*R^2^* = .12, *F*(1, 225) = 42.64, *p*< .001).

### 3. Using the online tool *statcheck*: a brief guide

*The statcheck* tool (Epskamp & Nuijten, 2016) is a free, open‐source R package with an accompanying Web application (statcheck.io). The package automatically extracts statistical results from papers and verifies their in‐text APA Style–reported NHST results. It is analogous to a spell checker for statistics. More package details and information on how to download it can be found on the CRAN package page (https://cran.r‐project.org/web/packages/statcheck/index.html).

#### 3.1 What does *statcheck* do?

The *statcheck* tool looks for results of NHSTs reported in APA Style (APA, 2010). It recognizes *t*‐tests, *F*‐tests, *χ2*‐tests, *Z*‐tests, correlations, and, with the updated version, *Q*‐tests (distinguishing among *Q*, *Q*‐between, and *Q*‐within tests). These types of tests consist of three elements: a test statistic, degrees of freedom, and a *p*‐value. These three elements are internally consistent; if you have two, you can calculate the third. The *statcheck* tool uses this characteristic to calculate if a reported *p*‐value matches the rest of the reported NHST result.

Take, for example, the following APA Style–reported NHST result: *t(14) = 1.23, p = .02*. If we use the degrees of freedom (14) and the test statistic (1.23) we can calculate that the *p*‐value of this result is .24. However, the reported *p*‐value was .02. This means that this result is *not* internally consistent. Furthermore, the reported *p*‐value is lower than the conventional level of significance of .05, meaning that this result would have been interpreted as significant. The recalculated *p*‐value, on the other hand, is not. The *statcheck* tool flags as “gross inconsistencies” or “decision errors” those results in which the reported *p*‐value is significant and the reported *p*‐value is not, or vice versa. By default, *statcheck* assumes an alpha level of .05.

Note that *statcheck* takes correct rounding into account. For instance, a reported *t*‐value of 2.35 could correspond to an actual value of 2.345 – 2.354 with a range of *p*‐values that can slightly deviate from the recomputed *p*‐value. The *statcheck* tool will not count cases like this as errors.

#### 3.2 Using the Web application

A quick and easy way to scan an article with *statcheck* is by using the Web application (statcheck.io).

First, decide whether to check the box “try to identify and correct for one‐tailed tests?” (see Figure A1). If checked, *statcheck* will consider results as correct if 1) the p‐value would have been consistent if the test was one‐tailed, and 2) if the words “one‐tailed,” “one‐sided,” or “directional” are somewhere in the full text.

Next, to upload an article to scan, click on the “Browse…” button (see Figure A1) and select an article in PDF, HTML, or DOCX format.

When an article has been selected, *statcheck* will automatically search it for APA Style–reported NHST results. The program extracts the results and recalculates the *p*‐values. The results will be displayed on the Web page (see Figure A2).

The results can be inspected online or downloaded in a CSV file by clicking the button “Download Results (csv)” (see Figure A2). The CSV file contains additional variables compared to the simpler web app output. Note that once all files have been analyzed, the source files are deleted. Outside of simple server and activity logs, no record of results is maintained.

#### 3.3 Interpreting the results returned by *statcheck*

Figure A2 shows the *statcheck* results for a single uploaded article. Each row in the data frame represents one extracted NHST result. The data frame consists of four columns. The column “Source” indicates the article from which the statistic on this particular line was extracted. Specifically, “Source” shows the file name of the uploaded article. In the example in Figure A2, both extracted statistics were from a file named “Paper1”.

The column “Statistical Reference” contains the complete statistical results that *statcheck* extracted from the paper. The statistical results consist of the reported test statistic, degrees of freedom (except in the case of z‐statistics), and *p*‐value. In the example, we see two extracted statistics: the results of a t‐test and the results of an F‐test.

In the next column, “Computed p Value”, you can find the *p*‐value that *statcheck* recalculated based on the reported test statistic and degrees of freedom. For example, in the first row of the example in Figure A2, a t‐test was extracted with 37 degrees of freedom and a t‐value of ‐4.93. Based on these two values, *statcheck* calculated that the accompanying *p*‐value is .00002.

Finally, the column “Consistency” indicates whether the recalculated *p*‐value is consistent with the reported *p*‐value (or, equivalently, whether the reported *p*‐value is consistent with the reported test statistic and degrees of freedom). There are three potential categories: Consistent, Inconsistency, or Decision Inconsistency. A result is classified as Consistent if the reported *p*‐value matches the recalculated *p*‐value. If the reported *p*‐value does not match the recalculated *p*‐value, but this would not change the statistical conclusion (i.e., both are significant or both are non‐significant), then the result is classified as an Inconsistency. If the reported and recalculated *p*‐value do not match and the statistical conclusion *would* change (i.e., the reported *p*‐value is significant and the recalculated *p*‐value is not, or vice versa), the result is classified as a Decision Inconsistency. In the first row of the example, the result is classified as Consistent: the reported *p*‐value (*p*< .001) is consistent with the recalculated *p*‐value (*p* = .00002). In the second row, the result is classified as a Decision Inconsistency: the reported *p*‐value was significant (*p*< .001) whereas the recalculated *p*‐value was not (*p* = .30793).

It is also possible that *statcheck* will not detect any APA Style–reported NHST results at all. In that case, the error message in Figure A3 appears.

The most common reason why *statcheck* does not find results is when results are not reported exactly according to APA Style. For *statcheck* to find a result, it needs to contain a test statistic, degrees of freedom, and p‐value, reported in that order. Another common issue is related to type‐setting problems in PDF files; some journals replace mathematical symbols such as “=” with images, and sometimes characters are encoded in a non‐standard way.

#### 3.4 Interpreting the *statcheck* output in the extended CSV file

As mentioned above, it is also possible to download the *statcheck* results in a more extensive CSV file. An example of what this output would look like is shown in Figure A4.

The first part of the output (see Figure A5) shows the statistics that were extracted from the uploaded article. Each row in the data frame represents one extracted NHST result.

Again, the column “Source” indicates the article from which the statistic on this particular line was extracted. Specifically, “Source” shows the file name of the uploaded article (in this case, “Paper1”).

The next columns (“Stat,” “df1,” “df2,” “Test Comparison,” “Reported Value,” “Reported Comparison,” and “Reported p Value”) show the full result that was extracted, separated by the different reported elements. Splitting up the different elements that were extracted can be useful if, for instance, you want to investigate how many F‐values an article or set of articles contains, or if you want to investigate the distribution of extracted p‐values.

The second part of the output (as shown below in Figure A6) shows information about the internal consistency of the extracted NHST result. The column “Computed p Value” shows the *p*‐value that *statcheck* calculated based on the reported test statistic and degrees of freedom. By default, *statcheck* assumes two‐sided tests, but if you checked the box “try to identify and correct for one‐tailed tests?,” it will try to do so.

In the column “Statistical Reference” are the complete results. This is the concatenated version of the information in the previous columns.

The columns “Error?” and “Decision error?” show the final results of the consistency check. If “Error?” is TRUE, it means that the computed *p*‐value does not match the reported *p*‐value and that this result seems to be an error. The same holds for “Decision error?”.

In this example, the first result shows that “Error?” is FALSE, which means that the reported p‐value is *not* an error, and that it is consistent with the reported test statistic and degrees of freedom. From this it also logically follows that “Decision error?” is FALSE for all these cases; a result can only be a decision error when it is an error in the first place.

The second row in Figure A6 illustrates an “Error?” and “Decision error?”. The column “Error?” is TRUE, which means that the reported p‐value is an error because it is not consistent with the reported test statistics and degrees of freedom. You can also see this in the output, where the reported p‐value is “< .001” (see “Statistical Reference”, rather than the rounded value in “Reported p Value”), whereas the computed *p*‐value is .31. This also means that “Decision error?” is TRUE. This error indicates that the statistical conclusion is incorrect: the reported *p*‐value is significant, whereas the *statcheck* recomputed *p*‐valueis not.

The third and last part of the output gives information about additional characteristics of the result and the uploaded article. The column “One‐sided testing?” indicates whether a result could have been a one‐tailed test by checking if the reported *p*‐value is exactly half of the *p*‐value that *statcheck* computed. If “One‐sided testing?” is TRUE, it means that the result would have been correct if it was a one‐tailed test.

The column “1‐tail in‐text” indicates whether the string “one‐tailed”, “one‐sided”, or “directional” was found in the full text that *statcheck* scanned. If “1‐tail in‐text” is TRUE, it could be a good indication that some or all of the tests are one‐tailed, which means that some of the flagged inconsistencies might not be inconsistent results at all.

If you did not check the box “try to identify and correct for one‐tailed tests?”, the columns “One‐sided testing?” and “1‐tail in‐text” can warn us when some of the errors that *statcheck* indicated could have been due to one‐tailed testing (Figure A7). In this example, we see that the words “one‐tailed,” “one‐sided,” or “directional” were mentioned in this article (“1‐tail in‐text” is TRUE in all cases). However, we also see that none of the extracted results would have been correct if they were in fact a one‐tailed test (“One‐sided testing?” is FALSE in all cases).

Finally, the column “APA factor” indicates the proportion of all detected *p*‐values that were part of a fully APA Style–reported NHST result. It gives a rough indication of how many statistical tests *statcheck* may have missed because of reporting issues. In our example, only 17% of all *p*‐values in the article were part of an NHST result fully reported in APA Style, so the statistics *statcheck* extracted are only a small sample of all statistics in the article.

#### 3.5 More information about *statcheck*

For more information about using the *statcheck* Web application, see the documentation at statcheck.io/documentation.php and the additional information at statcheck.io/about.php (see the tabs at the top of Web application).

For detailed information about how *statcheck* works, how to install and use the R package *statcheck*, and for any updates to the above manual of the Web application, see the extensive manual at hrpubs.com/michelenuijten/statcheckmanual.

For an example of how *statcheck* can be applied in documenting statistical reporting inconsistencies in large bodies of literature, and for detailed validity studies, see Nuijten et al. (2016; 2017).
